# A Red-Emitting, Multidimensional Sensor for the Simultaneous Cellular Imaging of Biothiols and Phosphate Ions †

**DOI:** 10.3390/s18010161

**Published:** 2018-01-09

**Authors:** Pilar Herrero-Foncubierta, Jose M. Paredes, Maria D. Giron, Rafael Salto, Juan M. Cuerva, Delia Miguel, Angel Orte

**Affiliations:** 1 Department of Physical Chemistry, Faculty of Pharmacy, University of Granada; Campus Cartuja, 18071 Granada, Spain; pilarhf@ugr.es (P.H.-F.); jmparedes@ugr.es (J.M.P.); dmalvarez@ugr.es (D.M.); 2 Department of Organic Chemistry, Faculty of Sciences, University of Granada; C. U. Fuentenueva s/n, 18071 Granada, Spain; jmcuerva@ugr.es; 3 Department of Biochemistry and Molecular Biology, Faculty of Pharmacy, University of Granada; Campus Cartuja, 18071 Granada, Spain; mgiron@ugr.es (M.D.G.); rsalto@ugr.es (R.S.)

**Keywords:** dual probes, fluorescent sensors, fluorescence lifetime imaging, FLIM, cellular stress, photoreceptor cells

## Abstract

The development of new fluorescent probes for cellular imaging is currently a very active field because of the large potential in understanding cell physiology, especially targeting anomalous behaviours due to disease. In particular, red-emitting dyes are keenly sought, as the light in this spectral region presents lower interferences and a deeper depth of penetration in tissues. In this work, we have synthesized a red-emitting, dual probe for the multiplexed intracellular detection of biothiols and phosphate ions. We have prepared a fluorogenic construct involving a silicon-substituted fluorescein for red emission. The fluorogenic reaction is selectively started by the presence of biothiols. In addition, the released fluorescent moiety undergoes an excited-state proton transfer reaction promoted by the presence of phosphate ions, which modulates its fluorescence lifetime, *τ*, with the total phosphate concentration. Therefore, in a multidimensional approach, the intracellular levels of biothiols and phosphate can be detected simultaneously using a single fluorophore and with spectral clearing of cell autofluorescence interferences. We have applied this concept to different cell lines, including photoreceptor cells, whose levels of biothiols are importantly altered by light irradiation and other oxidants.

## 1. Introduction

Intracellular sensing by using fluorescent probes is a well-established approach to monitor relevant biological processes at the cellular level. Understanding cellular function in terms of metabolism, differentiation, homeostasis, gene expression, or inter-cellular communication is a major aim for many interdisciplinary research branches, since these processes may be strongly affected by pathological states, such as neurodegenerative diseases or cancer [1,2]. Therefore, a thorough understanding of how diseases work at the molecular and cellular levels will provide an invaluable background to set the basis for new therapeutic tools. Nevertheless, the actual quantification of analytes or metabolites of interest has not been a trivial problem since the early days of immunofluorescence experiments [3]. The widespread use of fluorescent protein mutants [4] and advanced microscopy techniques, such as super-resolution nanoscopy [5], has resulted in a substantial boost of fluorescence-based cellular sensing.

An additional stimulating challenge in intracellular sensing is the capability of multiplexing; this is the simultaneous measurement of more than one parameter, which can provide important information on the correlation between cellular events and cause-effect relations. For the quantification of a single analyte, a widely accepted approach is based on ratiometric methods that use spectral separation in two channels for reconstructing ratio pseudo-images. However, intracellular multiplexing using ratiometric methods would require at least four different excitation/emission channels that make it a challenging problem that suffers from numerous complications and the lack of robustness, which limits their use. A powerful alternative to ratiometric methods for intracellular sensing is fluorescence lifetime imaging microscopy (FLIM) [6]. A group of fluorescent molecules that have been promoted to the excited state by a pulse of light will emit fluorescence, usually following exponential decay kinetics, whose decay rate (*k*) defines the so-called fluorescence lifetime (*τ*) as *τ* = 1/*k*, normally on the order of a few nanoseconds. FLIM microscopy exhibits many unique advantages for quantitative sensing compared to ratiometric fluorescence imaging, especially in terms of removing the contribution of cellular autofluorescence, a problem that may cause systematic errors in ratiometric methods [7]. Several FLIM-based intracellular sensors have been reported recently regarding the quantification of different analytes such as pH [8,9] or calcium [10], as well as other physical parameters such as temperature [11] or microviscosity [12,13]. Our research group has recently concentrated efforts on the development of different FLIM-based intracellular sensors [14,15]. Specifically, our thorough studies on the excited-state proton transfer (ESPT) reactions of xanthene derivatives, mediated by the presence of suitable proton acceptor/donor pairs [16,17,18], led us to propose a FLIM methodology and a family of sensors for the intracellular quantification of the total phosphate ions concentration [19,20]. The presence of a suitable proton donor/acceptor, such as the pair H_2_PO_4_^−^ and HPO_4_^2−^, does promote an inter-molecular proton transfer to the prototropic species of xanthene dyes. When this transfer occurs sufficiently rapid to compete with the fluorescence emission, it results in excited-state dynamics, thus altering the fluorescence emission properties of the dye [21,22]. The main parameters that define the ESPT reaction are: the total concentration of the proton donor/acceptor, the pH, and the excited-state acidity of the dye. Although this is not a specific feature of phosphate and other pairs can promote the reaction, such as acetate [16,23] and certain amino acids [24], not all the buffers are capable of promoting the ESPT reaction [25]. Interestingly, the family of xanthene derivatives so-called Tokyo Green dyes [26], which have the main feature of an off acidic prototropic form, showed fluorescence decay traces that were mostly mono-exponential when undergoing the buffer-mediated ESPT reaction, and the decay time of such fluorescence kinetics was dependent on the total phosphate concentration [17]. Based on these results, we started the development of fluorescent phosphate sensors in which the analytical parameter was the fluorescence lifetime of the dye, with particular usefulness for intracellular sensing using FLIM microscopy [19]. Phosphate ions are ubiquitous in many important processes such as energy storage, signal transduction, a myriad of phosphorylation/dephosphorylation reactions, and other processes such as osteoblast differentiation and bone deposition. Hence, the intracellular detection and quantification of these ions is a relevant matter for understanding cellular physiology [27,28,29].

Interestingly, the emission of fluorescent photons by electronically excited molecules is a multilayered, or multidimensional phenomenon. This process is defined by many different parameters that are orthogonal to each other, including the excitation and emission energy (colours), the emission intensity, the emission efficiency (quantum yield), the fluorescence lifetime (*τ*), and the polarization of the emitted light. This multidimensionality makes the fluorescence spectroscopy an extremely versatile tool, offering varied sources of information on the studied systems. Recently, we have taken advantage of the multidimensional character of the fluorescence emission process to develop an actual intracellular multiplexed sensing approach using a single dye sensor [30]. Our methodology consisted of a fluorogenic xanthene-based sensor that is reactive towards biological thiols, widely present in living cells but directly related to the presence of oxidative stress. Due to the high sensitivity of fluorescence techniques, the fluorogenic approach has been widely employed in biothiol sensing [31]. The novelty of our method is that the fluorescent molecule cleaved after the reaction with biothiols was a carefully selected xanthene derivative whose fluorescence lifetime value was dependent on the total phosphate ions concentration, which could be obtained through FLIM imaging. Therefore, by focusing on the fluorescence emission intensity, the thiol levels were accessible, whereas by inspecting the fluorescence lifetime of the release dye, the phosphate concentration was estimated. Other studies have shown the possibility of fluorogenic intracellular sensors for FLIM microscopy [32,33]; however, these sensors respond to a single analyte of interest. In contrast, our work exemplifies a novel and elegant use of multidimensional information to simultaneously report on two different analytes using a single sensor. In fact, the simultaneous estimation of these two parameters represents an invaluable tool to study dysfunctional cellular statuses, such as in obesity and diabetes, that exhibit alterations in bone metabolism with increased oxidative stress [34]. 

One of the advantages of FLIM microscopy for intracellular imaging is the possibility of discarding all interferences of cellular autofluorescence by applying a time-gated filtering method, by which the short-lived photons coming from cellular species are removed, leaving in the image only those photons arising from the species of interest [6]. However, this approach is more efficient when fluorophores exhibiting a long lifetime are employed. Unfortunately, one of the drawbacks of our xanthene derivatives to date is their spectral overlap with the cellular autofluorescence and their short lifetimes (usually <4 ns). This problem was addressed by including a lifetime contribution representing the cell autofluorescence and a second contribution for the specific dye. Although majorly corrected, some of the autofluorescence photons may be misplaced, causing an apparent decrease in the estimated lifetime, which leads to slightly overestimating the total phosphate concentration.

It is well known that the spectral region in which the cell autofluorescence is practically negligible is in the red and near-infrared (NIR) regions. Hence, the development of NIR fluorescent probes is currently a very active research field [35]. One of the most striking alternatives to achieve red-emitting dyes is the insertion of silicon atoms within the conjugated π moiety of the dye’s core. Silicon-substituted rhodamines [36,37], fluoresceins [38,39], and other xanthenes [40] have been reported in the literature as redshifted fluorophores for bioimaging probes. Interestingly, silicon-substituted fluoresceins, so-called Tokyo magenta (TM) dyes, still maintain the ability of undergoing an ESPT reaction mediated by the presence of the phosphate species present at a near-neutral pH [18]. A thorough investigation of the photophysics of 7-hydroxy-5,5- dimethyl-10-(*o*-tolyl)dibenzo[*b,e*]silin-3(5*H*)-one (2-Me TM), a dye of the Tokyo magenta family, described the kinetics of the excited-state reaction between the prototropic species of this dye and the H_2_PO_4_^−^/HPO_4_^2−^ pair as the proton donor/acceptor, reporting the values of all the kinetic rate constants involved in the reaction [18]. This reaction causes the fluorescence lifetimes of 2-Me TM to be dependent on the total phosphate concentration. Although this would be a primary condition to suggest this dye as an intracellular FLIM sensor of phosphate ions, 2-Me TM does not exhibit an on/off behaviour in its prototropic equilibrium, which results in complex bi-exponential decay kinetics of the fluorescence emission. This bi-exponential fluorescence decay dramatically hinders the usefulness of the dye as a FLIM sensor. Therefore, a clear step forward in the development of redshifted FLIM phosphate sensors entails the design of silicon-substituted xanthenes with on/off prototropic schemes.

In this work, we have synthesized a red-emitting, dual probe for multiplexed intracellular detection of biothiols and phosphate ions based on a fluorogenic construct involving silicon-substituted fluorescein, whose acidic form is eminently non-fluorescent and whose basic form is highly fluorescent. These prototropic features lead to virtually mono-exponential fluorescence decay traces, which can be phosphate-sensitive owing to the ESPT reaction. All these characteristics combine to represent a step forward with respect to our previous dual probe [30] because of the spectral clearing of cell autofluorescence interferences, which should provide a better response towards both biothiols and phosphate ions.

## 2. Materials and Methods

### 2.1. Synthesis: General Aspects

All reactions were performed in dry glassware and an air atmosphere. All commercially available solvents (dry dichloromethane (CH_2_Cl_2_), methanol (MeOH)) and reagents (4-bromo-3-methylanisole, *tert*-butyl litium solution, hydrochloric acid (HCl), 4-(dimethylamine)pyridine (DMAP) and 2,4-dinitrobenxenesulfonyl chloride) were used without further purification. For reactions involving organolithium derivatives, freshly distilled tetrahydrofuran (THF) in presence of Na wires and benzophenone was used. Thin-layer chromatography analysis was performed on aluminium-backed plates coated with silica gel 60 (230–240 mesh) with an F254 indicator. The spots were visualized with ultraviolet (UV) light (λ = 254 nm) and stained with phosphomolybdic acid solution and subsequent heating. NMR spectra were collected at room temperature at 400 MHz for ^1^H NMR and 101 MHz for ^13^C-NMR. Carbon multiplicities were assigned by DEPT techniques. HRMS were carried out by atmospheric-pressure chemical ionization (APCI+) or electrospray ionization (ESI).

The details of all the synthetic protocols, spectroscopic data and copies of NMR spectra of new compounds are shown in Appendix A.

### 2.2. Instrumentation

Absorption spectra were collected on a Lambda 650 UV-visible spectrophotometer (PerkinElmer, Waltham, MA, USA). Fluorescence emission spectra and kinetics were obtained on a Jasco FP-8300 spectrofluorimeter (Jasco, Tokyo, Japan), at the excitation wavelength λ_ex_ of 530 nm. Fluorescence quantum yields were obtained using Rhodamine 101 as a reference, as an average of 12 independent measures, of two different concentrations of the probe, two different concentrations of the reference, and three different excitation wavelengths. 

Images of the fluorescence emission intensities and fluorescence lifetimes were recorded on a MicroTime 200, fluorescence-lifetime microscope system (PicoQuant GmbH, Berlin, Germany). The excitation source consisted of a pulsed laser diode head pulsed laser at λ = 530 nm (LDH-P-FA-530B, PicoQuant, Berlin, Germany), operated by a PDL-800 driver (PicoQuant, Berlin, Germany) at a repetition rate of 20 MHz. The light beam was directed onto a dichroic mirror (Z532RDC, Chroma, Bellows Falls, VT, USA) to the oil immersion objective (100×, 1.4 NA) of an inverted microscope system (IX-71, Olympus, Tokyo, Japan). The fluorescence emission was directed to a 550-nm long-pass filter (AHF analysentechnik AG, Tübingen, Germany) and focused to a 75-µm pinhole. The fluorescence then passed through a bandpass filter (D630/60M, Chroma, Bellows Falls, VT, USA) and focused into a single-photon avalanche diode (SPCM-AQR 14, PerkinElmer). Imaging reconstruction, photon counting, and data acquisition were realized with a TimeHarp 200 TCSPC module (PicoQuant, Berlin, Germany). Raw images were obtained at a 512 × 512-pixel resolution over an area of 80 × 80 µm^2^. 

### 2.3. Cell Culture and Lysates

The human hepatocellular carcinoma HepG2 (ATCC no. HB-8065™) and MC3T3-E1 preosteoblasts (ECACC 99072810) cell lines were provided by the Cell Culture Facility, University of Granada. The mouse retinal cone-cell line 661W is a transformed cell line derived from mouse retinal tumours and was a gift from Dr. Enrique de la Rosa (CIB, CSIC, Madrid, Spain). HepG2 and 661W cells were grown at 37 °C in Dulbecco’s modified Eagle’s medium (DMEM) supplemented with 10% (v/v) fetal bovine serum (FBS), 2 mM glutamine plus 100 U/mL penicillin, and 0.1 mg/mL streptomycin. MC3T3-E1 cells were grown in alpha minimum essential medium (α-MEM) with ribonucleosides, deoxyribonucleosides, 2 mM L-glutamine and 1 mM sodium pyruvate, 10% (v/v) FBS and 100 U/mL penicillin, and 0.1 mg/mL streptomycin. 

For the FLIM microscopy experiments, the HepG2, MC3T3-E1 preosteoblasts and retinal cone 661W cell lines were seeded onto circular coverslips (diameter of 25 mm) in six-well plates at a density of 2.3 × 10^5^ cells per well.

For cell lysates, MC3T3-E1 cells were seeded at a density of 1 × 10^6^ cells/well in a p100 plate and incubated at 37 °C for 24 h to reach a cell confluence of 80–90%. Cells were washed twice with Hepes buffer 10 mM, pH 7.4, and scrapped in the same buffer. Cells were centrifuged at 800 × *g* for 10 min and the pellet of cells was resuspended in 100 μL of the Hepes buffer. The cell suspension was sonicated and centrifuged at 12,000 × *g* for 10 min. The supernatant was diluted 1:200, in the absence or the presence of 6 × 10^−4^ M of N-methylmaleimide (NMM), and DNBS-2Me-4OMe-TM was added to a final concentration of 6 × 10^−7^ M for measuring the fluorogenic response to cell lysates. 

### 2.4. FLIM Imaging Experiments

For both HepG2 and preosteoblasts cell lines, the reaction of DNBS-2Me-4OMe-TM with intracellular biothiols was initially followed. First, cells were washed twice with Krebs-Ringer buffer solution (118 mM NaCl, 5 mM KCl, 1.2 mM MgSO_4_, 1.3 mM CaCl_2_, 1.2 mM KH_2_PO_4_, 30 mM 4-(2-hydroxyethyl)-1-piperazineethanesulfonic acid (HEPES), at pH 7.4). The cover was then placed in the holder, and 1 mL of Krebs-Ringer buffer was added. Once the sample holder was placed in a microscope, the cellular autofluorescence was measured. The buffer solution was then removed and 1 mL of a 6 × 10^−7^ M solution of DNBS-2Me-4OMe-TM in the Krebs-Ringer buffer was added. Intensity images were collected every 5 min.

In a second step, once the biothiols had been measured, the fluorescence lifetime variation of the released 2Me-4OMe-TM with different amounts of phosphate ions was also analysed in the same cell lines. Since the plasma membrane is impermeable to phosphate ions, 1 µg/mL of α-toxin from *Staphylococcus Aureus* was added to the cells and incubated for 20 min. After this time, solutions of different total phosphate concentrations (10, 20, 30, 50 and 100 mM) were added. For the preparation of the phosphate buffer solutions, the individual phosphate species (NaH_2_PO_4_·H_2_O and Na_2_HPO_4_·7H_2_O; both from Fluka, puriss, p.a.) were mixed, and the pH was adjusted to 7.35. After adding each concentration, a FLIM image of the cell was collected. 

The cellular stress in photoreceptor cells was promoted by a preincubation with H_2_O_2_ (0-1 mM) for 12 h. Then, a similar protocol was used to measure the reaction of DNBS-2Me-4OMe-TM with biothiols. For the samples incubated with either 0.75 or 1 mM H_2_O_2_ cell death was observed before the FLIM measurements; thus, only results from the control (absence), and 0.25 and 0.5 mM concentrations are described.

All the data were analysed using the SymPhotime 32 (PicoQuant) package and the ImageJ distribution, FIJI [41]. Before the analysis, a 5 × 5 spatial binning was performed, for a final pixel size of 0.78 × 0.78 µm^2^, in order to increase the number of photons per pixel, to gain statistical robustness in the fitting of the fluorescence decay traces. The 2Me-4OMe-TM FLIM imaging was performed by fitting the fluorescence decay traces in each pixel to a bi-exponential decay function, using a reconstructed instrument response function (IRF) for the deconvolution analysis based on the maximum likelihood estimator. A short decay time of 1.5 ns was kept fixed to account for the contribution of the cell autofluorescence. The long decay time was an adjustable parameter, whose value was phosphate-dependent and assigned to the released 2Me-4OMe-TM. 

## 3. Results

**Scheme 1:**
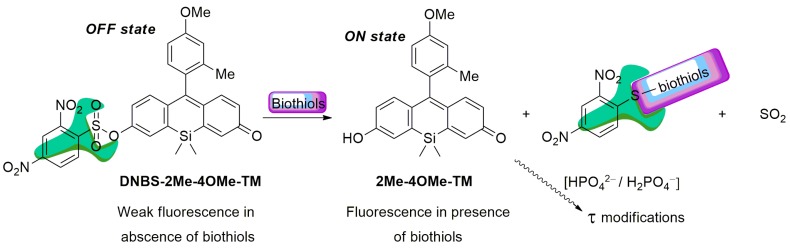
Working hypothesis of DNBS-2Me-4OMe-TM as a dual probe sensor for biothiols and phosphate ions (at a near neutral pH).

To achieve the objectives showed in the introduction, our new probe must be sensitive to both biothiol and phosphate concentrations in an independent but simultaneous way and exhibit emission in the red spectral range. To attain the dual sensing features, we previously used a highly fluorescent ON-OFF xanthene-based dye modified with a 2,4-dinitrobenzenesulfonic group (DNBS), susceptible to suffering a thiolysis reaction by biothiols present in the medium [30]. On the other hand, the substitution of the oxygen in the xanthone by a silicon atom can promote a bathochromic shift in the emission wavelength. The first precedent for this reaction was described by Maeda and colleagues [42]. In a first step, the sulphur nucleophile attacks at the aromatic ring thus releasing the fluorescent probe, sulphur dioxide and the (2,4-dinitrophenyl)biothiol sulphide (Scheme 1). Nevertheless, it is also known that, after the reaction, the (2,4-dinitrophenyl)biothiol sulphide can be hydrolysed in water, recovering a thiol group. This adds certain catalytic activity to the complex model of reaction. Thus, our working hypothesis consists of a weakly fluorescent compound that, in the presence of biothiols, will release a highly fluorescent dye, which in turn will respond to the phosphate ion concentration during its fluorescence lifetime (Scheme 1). In this sense, we prepared DNBS-2Me-4OMe-TM by the reaction of 2,4-dinitrobenzenesulfonyl chloride with the silicon-substituted xanthene 2Me-4OMe-TM, which was in turn prepared by a nucleophilic reaction of in situ prepared 2-methyl-4-methoxy-lithiobenzene to the corresponding silicon-substituted xanthone (See Appendix A for more details).

**Figure 1:**
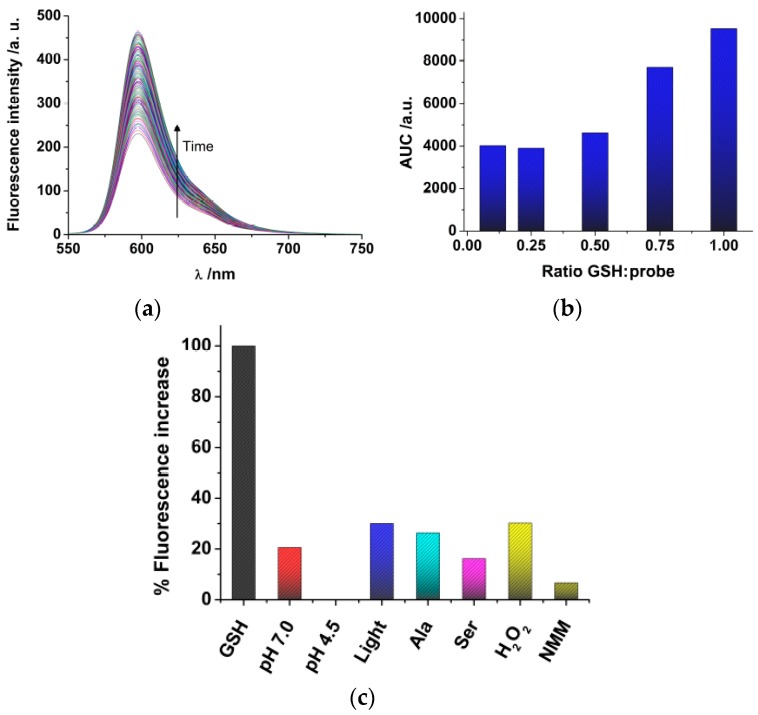
(**a**) Temporal evolution of the fluorescence emission spectra (λ_ex_ = 530 nm) of DNBS-2Me-4OMe-TM (6 × 10^−7^ M) in the presence of a stoichiometric amount of glutathione (GSH). (**b**) Area under the curve (AUC) of the response of DNBS-2Me-4OMe-TM (6 × 10^−7^ M) with different concentrations of GSH. (**c**) Stability and selectivity study of the fluorogenic reaction of DNBS-2Me-4OMe-TM (6 × 10^−7^ M).

Once we have synthesized DNBS-2Me-4OMe-TM, we tested the response towards glutathione (GSH), the most abundant source of thiols in live cells. Although free cysteine and homo-cysteine may be found in the cell cytoplasm, their relative abundance is usually lower than 10% that of GSH; hence, we focused our experiments in solution on the response of the probe to GSH. Once the DNBS-2Me-4OMe-TM probe is dissolved in the presence of GSH, the emission intensity shows a time-dependent increase (Figure 1a) due to the release of 2Me-4OMe-TM. To quantify the extent of the fluorogenic reaction, we employed the area under the curve (AUC) of the emission intensity at the emission maximum, 597 nm, with λ_ex_ = 530 nm. We also obtained other spectroscopic features of both the DNBS-2Me-4OMe-TM and the 2Me-4OMe-TM to ensure the fluorogenic nature of the reaction. Whereas the DNBS-2Me-4OMe-TM exhibited absorbance and emission values within background levels, at the experimental conditions, the 2Me-4OMe-TM dye showed an absorbance maximum at 583 nm, with molar absorptivity of 165,300 M^−1^ cm^−1^, and a fluorescence quantum yield of 0.44 ± 0.03. We then tested the response of the DNBS-2Me-4OMe-TM probe to different concentrations of GSH (Figure 1b). Using as a reference the AUC of a molar ratio [GSH]/[probe] = 1, the decrease to a ratio of 0.75 caused a 19% decrease in the signal (representing a signal of 0.81). Likewise, a [GSH]/[probe] molar ratio of 0.5 resulted in a decrease in the reference signal of 51% (representing a signal of 0.49). This means that linearity is well fulfilled in such concentration ranges. However, when the GSH concentration was further decreased, the response was no longer linear, reaching a background limiting value. This suggests that a potential interfering reaction may be causing the hydrolysation of the probe, and the release of 2Me-4OMe-TM.

We then performed a study of the stability and selectivity of the DNBS-2Me-4OMe-TM probe (Figure 1c). Using as a reference the AUC of the reaction with GSH, we first explored the stability of the probe, in the absence of thiols, in aqueous solution at pH 7.0 and 4.5 (the latter being the pH in the cellular lysosomes). Importantly, we found release of 2Me-4OMe-TM to a certain extent (20.6% of the signal with GSH) at pH 7.0, whereas the DNBS-2Me-4OMe-TM was totally stable at pH 4.5, exhibiting no fluorescence increase. Given that the DNBS group is similar to those employed for photo-uncaging, fluorogenic reactions, such as α-carboxy nitroveratryl [43,44] and other *o*-nitrobenzyl protecting groups [45], one could think on the spontaneous photolysis of the DNBS group as the cause for the release of 2Me-4OMe-TM at pH 7.0. However, the photolytic uncaging of such groups requires light in the near-UV spectral region, below 420 nm. Hence, it is not likely that a lower energy radiation, such as that used in our experiments (530 nm), was capable of producing the photo-uncaging effect. Despite this, we also explored the effect of white light irradiation of the DNBS-2Me-4OMe-TM probe, and found an increase in the fluorescence emission of 30.1% (referred to the presence of GSH). Therefore, photolysis of the DNBS group, caused by external light, may have certain weight on the stability of the off probe DNBS-2Me-4OMe-TM. Moreover, we studied other potential interferences that could cause a fluorogenic response of the probe, such as oxidants (H_2_O_2_) and nucleophile amino acids (alanine, Ala; and serine, Ser). The amino acids showed a response similar to that of the probe alone at pH 7.0, whereas H_2_O_2_ caused a hydrolysation similar to that of the irradiated sample (30.2%), meaning that these are not causing any additional interference than that already in place. We also found that N-methylmaleimide (NMM) exhibited a protective effect, so that the fluorogenic dye was less hydrolysed at pH 7.0.

Finally, before applying the probe to the intracellular environment, we investigated the behaviour of the DNBS-2Me-4OMe-TM fluorogenic reaction in cell lysate, blocking the GSH and other thiols with NMM. With this experiment, we can identify other sources of interference in the cell cytoplasm. We obtained that when the thiols were blocked with NMM the AUC of the release of 2Me-4OMe-TM was 24% of that response in cell lysate in the absence of NMM. This means that the endogenous thiols in the cell extract were effectively blocked, and that other potential factors of interference have a similar effect to those already described above. Hence, the experiment confirms the selectivity of the probe towards thiols, although not yet totally specific.

**Figure 2:**
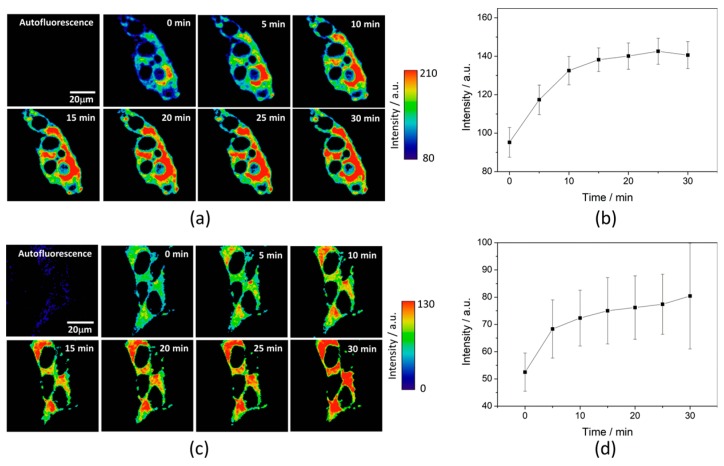
Fluorogenic reaction of DNBS-2Me-4OMe-TM (6 × 10^−7^ M) in response to intracellular biothiols. (**a**) Representative example of fluorescence intensity imaging with time in HepG2 cells, and (**b**) the emission intensity averaged over the pixels in the cytoplasm of 10 repeated experiments containing several cells. Error bars represent the standard errors. (**c**) Representative example of fluorescence intensity imaging with time in the preosteoblasts, and (**d**) the emission intensity averaged over the pixels in the cytoplasm of five repeated experiments. Error bars represent the standard errors.

**Figure A1:**
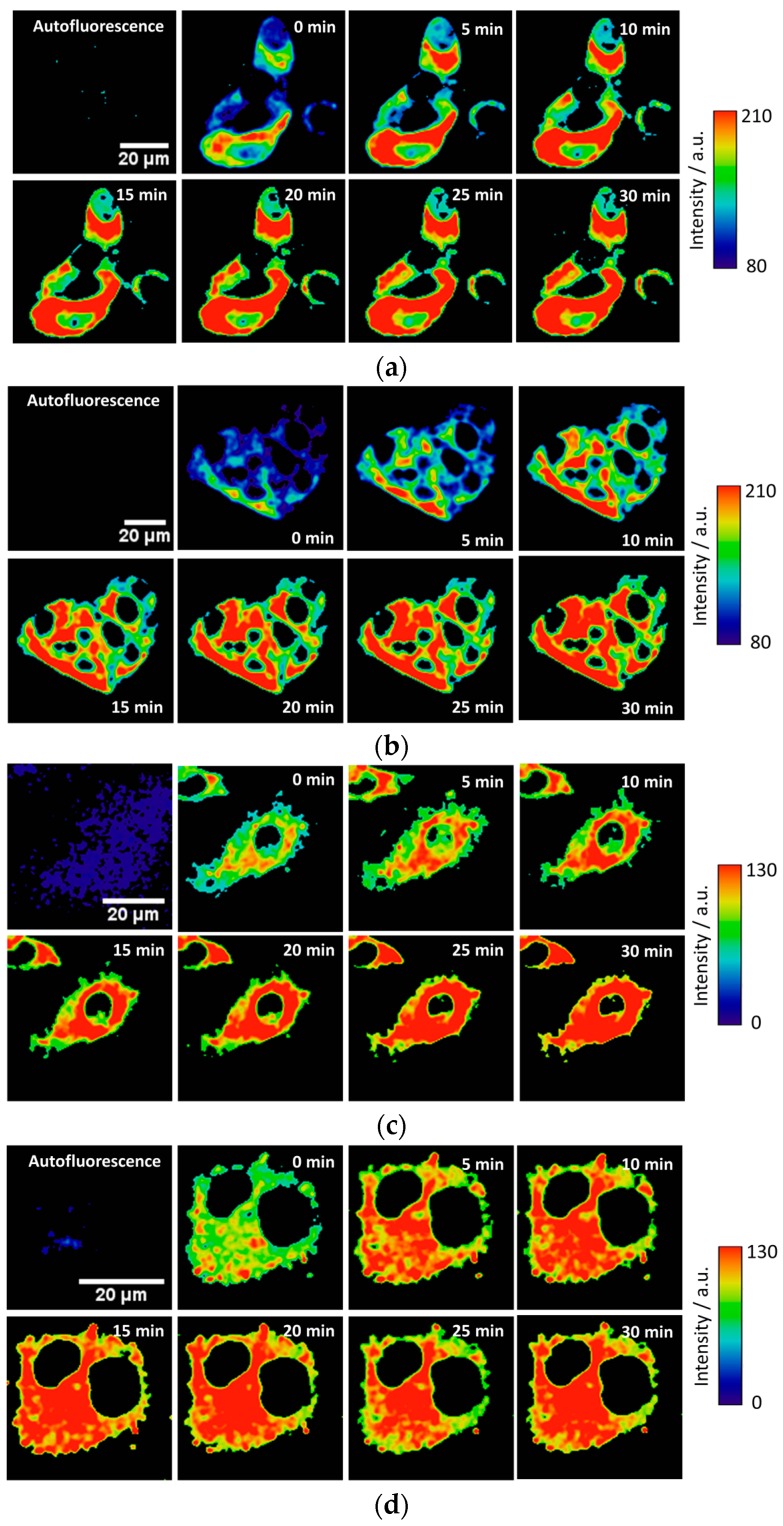
Other representative examples of the fluorogenic reaction of DNBS-2Me-4OMe-TM (6 × 10^−7^ M) in response to intracellular biothiols in (**a**,**b**) Hep2G and (**c**,**d**) MC3T3-E1 cells. Only pixels in the cytoplasm are shown.

To test the intracellular performance of the DNBS-2Me-4OMe-TM dual probe, it was added to the extracellular medium of HepG2 and preosteoblast cells, resulting in the spontaneous and fast incorporation of the probe inside the cells. Immediately, the thiolysis reaction started releasing the fluorescent moiety 2-Me-4OMe-TM, thus increasing the intensity with time, as seen in Figure 2a,c, where images of the two cell lines are represented (additional examples can be found in Figure A1 in Appendix B). As observed, the fluorescence emission exhibits higher values and faster kinetics in HepG2 cells than it does in the preosteoblasts, suggesting higher levels of biothiols in the former. Figure 2b,d represent the kinetics of the average intensity using different images from different experiments of HepG2 and preosteoblast cell lines, respectively.

**Figure 3:**
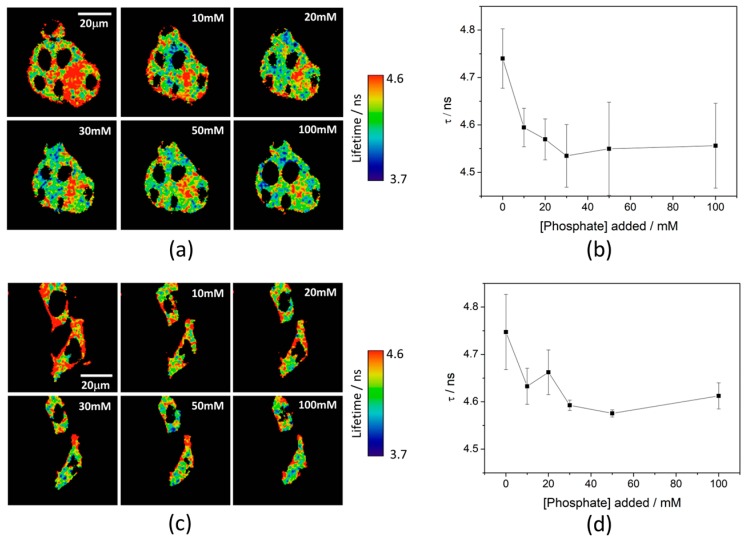
Phosphate response of the released 2Me-4OMe-TM in fluorescence lifetime imaging microscopy (FLIM) imaging. (**a**) Representative FLIM images of released 2Me-4OMe-TM in HepG2 cells, incubated with α-toxin for 20 min at different extracellular phosphate concentrations, and (**b**) the corresponding fluorescence lifetime *τ* in the cytoplasm pixels averaged over 10 repeated experiments. Error bars represent the standard errors. (**c**) Representative FLIM images of released 2Me-4OMe-TM in preosteoblast cells, incubated with α-toxin for 20 min at different extracellular phosphate concentrations, and (**d**) the corresponding fluorescence lifetime *τ* in the cytoplasm pixels averaged over four repeated experiments. Error bars represent the standard errors.

**Figure A2:**
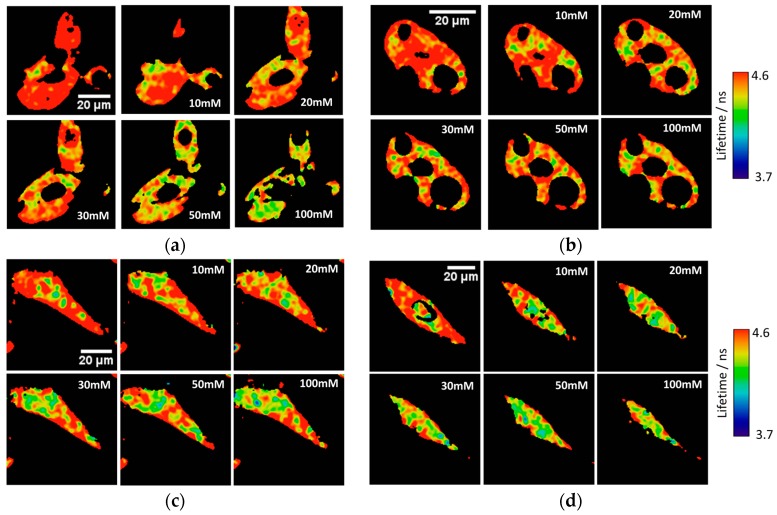
Other representative examples of the variation of the phosphate response of the released 2Me-4OMe-TM in FLIM imaging in (**a**,**b**) Hep2G and (**c**,**d**) MC3T3-E1 cells, permeabilised with α-toxin and different extracellular phosphate concentrations.

Simultaneously, and by focusing on the fluorescence lifetime, *τ*, of the released 2Me-4OMe-TM, the intracellular phosphate concentration can be estimated. As it is described by the theory of buffer-mediated ESPT reactions [21,22], the fluorescence lifetime is dependent on the presence of an adequate proton/donor acceptor as the phosphate buffer. For this ESPT reaction to occur, the 2Me-4OMe-TM must undergo an acid-base equilibrium with a p*K_a_* value that, optimally, matches that of the proton donor/acceptor buffer. Indeed, we obtained that the 2Me-4OMe-TM has a neutral, non-emissive form, whose molar absorptivity was 12,300 M^−1^ cm^−1^ at 499 nm, and a ground-state p*K_a_* of 7.26 ± 0.03. To test the capability of the released 2Me-4OMe-TM to report on the intracellular phosphate ions concentration, we opened small pores in the cell membrane using α-toxin, allowing small molecules (such as phosphate ions) to enter and exit freely from the medium to the cell without any loss of high molecular weight cellular compounds. Next, we obtained FLIM images using different extracellular buffers with different phosphate concentrations. Figure 3a,c show FLIM images of the released 2Me-4OMe-TM in HepG2 and preosteoblast cell lines at different phosphate concentrations. Using the arbitrary colour scale (from 4.6 to 3.7 ns) to represent the fluorescence lifetime, it is possible to directly notice a decrease in the *τ* values with the addition of phosphate. We repeated the experiment with 10 (for HepG2) and five (for preosteoblasts) different sets of cells (additional examples can be found in Figure A2 in Appendix B), to obtain the average *τ* values as a function of the total phosphate concentration (Figure 3b,d). The fluorescence lifetime exhibits a marked decrease with phosphate concentration up to approximately 50 mM, as the ESPT reaction theory predicts. Higher phosphate concentrations did not cause any further decrease in the *τ* values. These results combined with those obtained in the fluorogenic thiolysis of DNBS-2Me-4OMe-TM support the capability of this probe to simultaneously determine amounts of both biothiols and phosphate anions in cell media.

**Figure 4:**
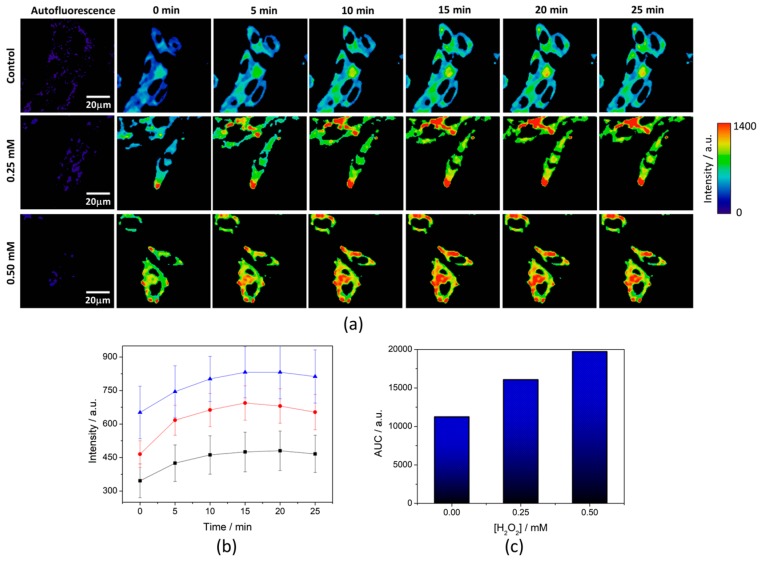
(**a**) Fluorescence intensity images of DNBS-2Me-4OMe-TM (6 × 10^−7^ M) in 661W cells after 12 h incubation with 0, 0.25, and 0.50 mM of H_2_O_2_, and (**b**) the corresponding emission intensity averaged over the pixels in the cytoplasm of at least four repeated experiments containing several cells (H_2_O_2_ concentration: 0 mM, black; 0.25 mM, red; 0.50 mM, blue). Error bars represent the standard errors. (**c**) AUC values for the data are shown in panel (**b**).

Finally, we tested the probe in the 661W cell line, previously used as model of the photoreceptor cells [46]. Multiple diseases related with the degeneration of the retina are caused by high oxidative stress generated in the photoreceptor cells. As an indirect application to detect cellular stress through biothiols, we incubated 661W cells for 12 h with different concentrations of hydrogen peroxide (H_2_O_2_). To fight against the cellular stress generated by H_2_O_2_, these cells synthesize higher concentrations of glutathione and other biothiols. This behaviour can be observed in the illustrative examples given in Figure 4a in which the fluorogenic reaction of DNBS-2Me-4OMe-TM is shown in the control cells (without H_2_O_2_ addition), and in those incubated with 0.25 mM and 0.5 mM of H_2_O_2_. These images clearly illustrate that not only is the intensity increase faster, but also it achieves a higher value in the presence of H_2_O_2_. In Figure 4b, the intensity curves at different reaction times (averaged over different experiments) are shown. Finally, Figure 4c represents the AUC calculated from all the measurements from Figure 4b. As is observed, the AUC values can be related to the presence of intracellular biothiols generated by cellular stress.

## 4. Discussion and Conclusions

The simultaneous detection of different analytes is an important concept in the study of live cell physiology and pathology because it allows the direct correlation of homeostatic responses as well as the cause-effect relations. Indeed, multiplex detection in live cells is a key approach to the study of physiological pathways and metabolic mechanisms in which studied analytes are involved. In this work, we have tested the effectiveness as a dual probe for the simultaneous detection of intracellular phosphate and biothiols. Following a recent methodology [30], a new compound was designed to combine some appropriate characteristics for the analysis in live cells. First, the use of red fluorescence (λ_em_ = 595 nm) as an analytical signal has great interest in biological measurements because it allows minimizing the interferences that come from natural green autofluorescence. The redshift is caused by the incorporation of a Si atom instead of O in position 10 of a xanthene moiety [38,40]. Second, the fluorophore was specifically designed to preserve a fast and spontaneous incorporation inside the cells due to its low hydrophilic character [18,19,26]. As biothiol detection involves the incorporation of the dinitrobenzenesulfonate (DNBS) group to the fluorophore, we checked that the increase in size of the molecule and the incorporation of the Si atom do not alter the natural membrane permeability of this dye. The acquired images demonstrate excellent cell incorporation, as shown in Figure 2, Figure 3 and Figure 4.

The sensor that we describe herein can act as a real-time, dual probe. The simultaneous detection is based on the measurement of two different parameters; for biothiols, we used the increase in intensity due to the thiolysis of the DNBS-2Me-4OMe-TM releasing the high fluorescent moiety 2Me-4OMe-TM [30]. In contrast, the phosphate determination is based on the changes in the fluorescence lifetimes due to an ESPT reaction promoted by the phosphate ions [18,21]. The presence of phosphate in the medium makes the ESPT reaction faster than the fluorescence process and consequently, it produces a decrease in the fluorescence lifetime. This decrease is dependent on the phosphate concentration and allows us to determine the intracellular phosphate flux.

To asseverate that the compound responds correctly to both analytes, we introduced it into two different cell lines (HepG2 and MC3T3-E1 cells). We monitored the fluorescence intensity increase mediated by biothiols through the thiolysis reaction. As seen in Figure 2, DNBS-2Me-4OMe-TM has a sensitive response to the intrinsic biothiols, avoiding potential issues of cellular autofluorescence due to spectral separation. As previously described, the sensor is based in the quenching produced by the DNBS group by photoinduced electron transfer [42]. A well-described thiolysis reaction can release the attached fluorophore, producing an increase in fluorescence. This strategy has been previously employed in other thiol probes [42,47,48,49,50]. Similar to other DNBS-based probes, DNBS-2Me-4OMe-TM presents a rapid intracellular response, where biothiol levels can be assessed in approximately 20 min. using a semi-quantitative approach through the AUC method. The AUC method of the fluorescence kinetics has become an adequate parameter to estimate the biothiol levels synthesized in cells due to oxidative stress. Although several studies demonstrated the intracellular use as biothiol sensors of other DNBS-based molecules [47,50,51], these studies did not perform an intracellular semi-quantitative estimation of biothiol levels. 

Once the fluorogenic reaction was finished, with the use of α-toxin from *Staphylococcus aureus*, we generated membrane pores that allow the diffusion of low molecular weight molecules, such as phosphates, and we added increasing phosphate concentrations to the medium, thus reaching an equilibrium between the intra- and extracellular phosphate concentrations. Fluorescence lifetime images in Figure 3 were acquired at different phosphate concentrations, and they show that the fluorescence lifetime of the released 2Me-4OMe-TM is sensitive to intracellular phosphate concentrations, as the ESPT reaction theory predicts [21]. The obtained *τ* values exhibit a slight decrease with the phosphate concentration. Although this decrease is enough to determine the intracellular phosphate concentration, its dependency is less marked than that obtained with other xanthenic derivatives. 

This multiplex detection of biothiols jointly with the phosphate ions, based on the multiparametric character of the fluorescence emission, is the distinctive feature of our approach. To the best of our knowledge, only our previous biothiol/phosphate probe is reported [30] in the literature, but as a novelty, in this work, we have focused on the optimization for an actual intracellular application. For this purpose, we have shifted the fluorescence from green to red and reduced the time to measure intracellular biothiols from 60 to 20 min., and the lifetime sensitivity has been improved at a low phosphate concentration (approximately 0.2 ns with the addition of 30 mM of phosphate). However, at a higher phosphate concentration (approximately >50 mM), the probe loses its sensitivity. The main reason behind this lower response may come from the extra hydrophobicity that the silicon atom confers to the molecule. This effect may induce strong interactions with the intracellular membranous compartments, keeping the molecule protected from the phosphate proton donors/acceptors that would promote the ESPT reaction. We are currently investigating the effect of microheterogeneous systems, such as micelles, to the phosphate-mediated ESPT reaction of the 2Me-4OMe-TM dye. These studies will shed some light on the excited-state behaviour of the dye and will allow us to have a better control over the ESPT reaction, thus providing invaluable information for a rational design of further modifications to red-emitting silicon-fluoresceins as phosphate sensors.

To consolidate the multiplexing approach of our method, we have analysed the lifetime images of the experiments shown in Figure 2 and Figure A1. These images show the increase in the fluorescence intensity with time upon release of the 2Me-4OMe-TM dye. When we analysed the associated FLIM images, we obtained average lifetimes of the 2Me-4OMe-TM dye in the images of HepG2 and MC3T3-E1 cells. By interpolating these values into the calibration curves depicted in Figure 3, we quantified the intracellular phosphate levels to be 1.9 ± 1.1 mM in the HepG2 cells and 4.2 ± 2.1 mM in the MC3T3-E1 preosteoblasts. These values are in good agreement with the typical concentration of free phosphate ions in the cell cytoplasm, dynamically kept around 10 mM, in the absence of specific alterations or signalling events [52]. The large associated error may come from the fact that, at least, 20 different cells in homeostatic equilibrium and different physiological stages are probed. Likewise, pH level variations between the different cellular compartments may contribute to the broadening of lifetime distributions, and hence, to increase the width of the distribution of total phosphate concentration values. 

Once the performance of the DNBS-2Me-4OMe-TM dye as a dual probe was confirmed, we planned its use in biological applications of interest. In this sense, we paid attention to the oxidative stress consequences over cellular homeostasis. Specifically, we focused on the loss of vision generated by oxidative stress in the photoreceptor cells in age-related macular degeneration (AMD) or diabetic retinopathy [53,54,55,56]. For this purpose, we induced oxidative stress in 661W cells (a mouse-derived photoreceptor cell line) by adding different concentrations of H_2_O_2_ during 12 h of incubation. The oxidative stress generated produced an increase in the intracellular biothiol synthesis as a protective cell response. After the 12-h incubation, we added DNBS-2Me-4OMe-TM and measured the fluorogenic response to the biothiol levels. The results obtained (Figure 3) show a direct dependence between the increase in fluorescence and the oxidative stress generated. The presence of reactive oxygen species (ROS) produces a higher intracellular biothiols synthesis and thus a faster and larger increase in the fluorescence intensity. These findings suggest that this technique is a direct methodology that can be used to develop high-throughput tests for ROS generating molecules and that it may easily be extended to study antioxidant drugs.

## Figures and Tables

**Scheme A1:**
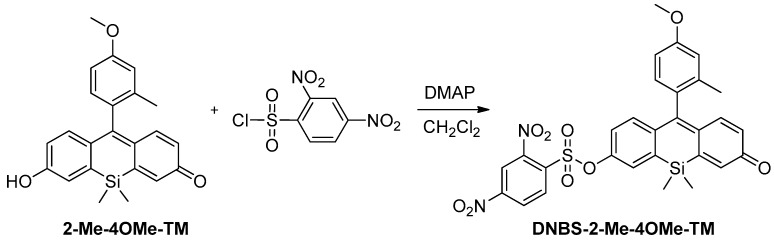
Synthesis of DNBS-2Me-4-OMe-TM.

**Scheme A2:**
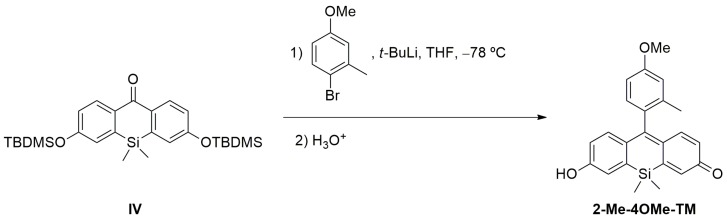
Synthesis of 2Me-4-OMe-TM.

**Scheme A3:**
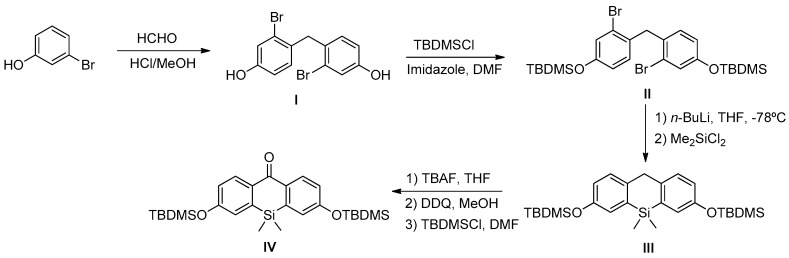
Synthesis of precursor **IV**.

## Appendix A. Synthesis of DNBS-2Me-4OMe-TM

### Synthesis and Spectroscopic Data of Compounds DNBS-2Me-4OMe-TM and 2-Me-4OMe-TM

Compound DNBS-2-Me-4OMe-TM was synthesized by nucleophilic addition of 2Me-4OMe-TM to 2,4-dinitrobenzenesulfonic acid in basic media and CH_2_Cl_2_ as solvent (See Scheme A1). Thus, 4-(dimethylamine)piridyne (24 mg, 0.196 mmol) was added to a solution of 2Me-4OMe-TM (49 mg, 0.131 mmol) in dry CH_2_Cl_2_. Then, 2,4-dinitrobenzenesulfonyl chloride (42 mg, 0.157 mmol) was added. After 10 min. stirring at room temperature, the solvent was removed under reduced pressure, and the residue was purified by column chromatography with CH_2_Cl_2_/MeOH mixtures as eluent, giving the corresponding dinitrobenzenesulfonate derivative as a yellow-orange solid (44.4 mg, 56%).

The spectroscopic data of compound DNBS-2Me-4OMe-TM are as follows: **^1^H NMR (400 MHz, Acetone-d_6_)** δ 8.97 (d, *J* = 2.3 Hz, 1H); 8.73 (dd, *J* = 8.7, 2.3 Hz, 1H); 8.38 (d, *J* = 8.7 Hz, 1H); 7.63 (d, *J* = 2.8 Hz, 1H); 7.19 (dd, *J* = 8.9, 2.8 Hz, 1H); 7.07–6.96 (m, 4H); 6.94 (dd, *J* = 8.3, 2.6 Hz, 1H); 6.81 (d, *J* = 2.2 Hz, 1H); 6.18 (dd, *J* = 10.2, 2.2 Hz, 1H); 3.88 (s, 3H); 2.00 (s, 3H); 0.49 (s, 3H); 0.47 (s, 3H). **^13^C NMR (101 MHz, Acetone-d_6_)** δ 184.2 (C), 160.87 (C), 153.2 (C), 150.1 (C), 146.8 (C), 141.9 (C), 141.8 (C), 141.6 (CH), 138.4 (CH), 135.3 (CH), 134.8 (CH), 133.1 (C), 132.1 (C), 131.7 (C), 131.3 (CH), 129.2 (CH), 128.4 (CH), 128.1 (CH), 124.5 (CH), 121.8 (CH), 116.6 (CH), 112.4 (CH), 55.7 (CH_3_), 19.9 (CH_3_), −1.5 (CH_3_), −1.9 (CH_3_). **HRMS (ESI):** [M + H]^+^ calcd. for C_29_H_25_N_2_O_9_SSi: 605.1050, obtained: 605.1040.

Compound 2-Me-4-OMe-TM was prepared by nucleophilic addition of the organolithium reagent generated by the halogen-lithium exchange reaction of 4-bromo-3-methylphenol with *t*-BuLi to the silicon-substituted ketone **IV**. Then, the corresponding alcohol obtained underwent a dehydration reaction by acidic hydrolysis, giving Tokyo Magenta derivative (see Scheme A2). In this sense, 4-bromo-3-methylanisole (100 mg, 0.497 mmol) was dissolved in dry THF under an Ar atmosphere in a Schlenck tube, and the solution was cooled to −78°C. Then, *tert*-butyllitium solution (0.58 mL, 0.995 mmol) was added at low temperature, and after stirring 30 min. at –78°C, a solution of ketone **IV** (124 mg, 0.249 mmol) in dry THF (2 mL) was slowly added. The solution was stirred at low temperature for 20 min. and then let warm to room temperature. After one hour at room temperature diluted HCl (1 mL, 10% solution) was added to the reaction. The solvent was then removed under low pressure, and the residue was purified by column chromatography using CH_2_Cl_2_/ MeOH mixtures as eluent. 2Me-4OMe-TM was obtained as a pink solid (55 mg, 59.14%), giving the following spectroscopic data: **^1^H NMR (400 MHz, Methanol-d_4_)** δ 7.02 (d, *J* = 2.5 Hz, 2H); 7.00 (s, 1H), 6.98 (s, 2H); 6.95–6.90 (m, 2H); 6.46 (d, *J* = 2.5 Hz, 1H); 6.44 (d, *J* = 2.5 Hz, 1H); 3.87 (s, 3H); 2.01 (s, 3H); 0.49 (s, 3H); 0.48 (s, 3H). **^13^****C NMR (101 MHz, Methanol-d_4_)** δ 162.0 (C), 161.3 (C), 154.1 (C), 154.0 (C), 141.30 (CH), 141.26 (CH), 138.6 (C), 132.7 (C), 131.4 (CH), 129.7 (C), 122.7 (CH), 116.5 (CH), 112.3 (CH), 55.8 (CH_3_), 19.9 (CH_3_), −1.3 (CH_3_), −1.6 (CH_3_). **HRMS (APCI+):** [M+H]^+^ calcd. for C_23_H_23_O_3_Si: 375.1411, obtained: 375.1424.

Finally, compound **IV** was synthesized according to the protocol described by Best et al. [57]. Thus, condensation of 5-bromophenol with formaldehyde gave compound **I**, whose hydroxyl groups were protected as *tert*-butyldimethylsilane (TBDMS) groups. Treatment of **II** with *n*-BuLi at −78°C followed by chlorodimethylsilane addition led to compound **III**. Then, the deprotection of TBDMS groups and later methylene group oxidation gave rise to the corresponding ketone group at the 9 position of the silicon-substituted xanthene. Finally, precursor **IV** was obtained after a double protection of hydroxyl groups as TBDMS (Scheme A3). Spectroscopic data of compounds **I**-**IV** were identical to those previously described [57].
